# Metagenomics: A New Way to Illustrate the Crosstalk between Infectious Diseases and Host Microbiome

**DOI:** 10.3390/ijms161125957

**Published:** 2015-11-03

**Authors:** Yinfeng Zhang, Cheuk-Yin Lun, Stephen Kwok-Wing Tsui

**Affiliations:** 1 School of Biomedical Sciences, The Chinese University of Hong Kong, Hong Kong, China; yinfengzh@cuhk.edu.hk (Y.Z.); yinyinlun@yahoo.com.hk (C.Y.L.); 2 Hong Kong Bioinformatics Centre, The Chinese University of Hong Kong, Hong Kong, China; 3 Centre for Microbial Genomics and Proteomics, The Chinese University of Hong Kong, Hong Kong, China

**Keywords:** microbiome, metagenomics, next generation sequencing, infectious disease, HIV, HBV, influenza, tuberculosis

## Abstract

Microbes have co-evolved with human beings for millions of years. They play a very important role in maintaining the health of the host. With the advancement in next generation sequencing technology, the microbiome profiling in the host can be obtained under different circumstances. This review focuses on the current knowledge of the alteration of complex microbial communities upon the infection of different pathogens, such as human immunodeficiency virus, hepatitis B virus, influenza virus, and *Mycobacterium tuberculosis*, at different body sites. It is believed that the increased understanding of the correlation between infectious disease and the alteration of the microbiome can contribute to better management of disease progression in the future. However, future studies may need to be more integrative so as to establish the exact causality of diseases by analyzing the correlation between microorganisms within the human host and the pathogenesis of infectious diseases.

## 1. Introduction

Nowadays, “microbiome” is used as a term to describe the whole genetic information of a microbial complex, especially referring to an environmental sample that contains different kinds of microbes. More specifically, the human microbiome could be considered as the collection of genomes of bacteria, viruses, fungi, and archaea inhabiting in and on humans. However, it seems that the term microbiome is commonly used to describe the bacterial population only. Therefore, sometimes the populations of viruses and fungi are described as virome and mycobiome, respectively.

Over the past few years, the study of microbiome has undergone a veritable explosion of growth. Considering the fact that nearly 90% of the cells in and on the human body are microbial cells and the vast numbers of symbiotic microbes contribute 100 times more genes than humans, the human body could be regarded as a “house” or “scaffold” for living organisms. To date, the normal microbiome of several body parts have been described, including the gut [1], oral cavity [2], conjunctiva [3], respiratory tracts [4], urogenital/vaginal tract [5], skin [6], and even in the blood stream, which was previously considered as a sterile environment [7].

One major obstacle for better recognizing and understanding the microbial world is that, although the taxonomic diversity of microbes is remarkably rich, 90% of them could not be cultivated in the laboratory [8]. Recent advances in the next generation sequencing, which does not require culturing and prior knowledge of pathogens, has brought a revolution in research and clinical applications in the field of the microbiome. With the huge amount of data and modest cost, it is now much easier to obtain the information for identification of microbes and their diversity, evolution, transmission, pathogenesis, and drug resistance. Three major metagenomic sequencing techniques, namely 16S rRNA sequencing, whole genome sequencing, and shotgun metagenomic sequencing, are applied for different purposes. The 16S rRNA sequencing is the first molecular tool aimed at the highly-conserved 16S ribosomal gene for accurate identification of bacterial isolates and discovery of novel bacteria. However, the 16S rRNA could not be used to classify virus or eukaryotic microbes. In contrast, to understand an organism comprehensively, whole genome sequencing is a more powerful tool. The first complete genome sequence of *Haemophilus influenzae* was reported in 1995 [9]. Now, thousands of complete bacterial genomes have been deposited in public nucleotide databases. Last but not least, the shotgun metagenomic sequencing can be used to sequence total DNA of a microbial community simultaneously. This method helps identify organisms without a phylogenetic marker gene, such as viruses or other eukaryotic components. More importantly, it can be used to tell the functions of the microbes. Recent reports also suggested that these methods were applied in identifying abnormal microbiota in humans contributing to Type 2 diabetes and atherosclerosis [10,11]. With the new techniques, human microbiome have been massively characterized through large-scale sequencing projects, such as the Human Microbiome Project (HMP), conducted by the US National Institutes of Health and the Metagenomics of the Human Intestinal Tract project (MetaHIT) hosted by the European Commission. The ultimate goal of these projects is to improve human health through the modulation of the human microbiome.

Previously, the human microbiota are often taken as commensals; yet they can be engaged in mutual interactions with their hosts, directly or indirectly. The microbiome has been described as an ecosystem in which equilibrium of various members is required to keep a person healthy [12]. The colonization resistance phenomenon of healthy gut microbiota is a well-known example to help host defense against the pathogen infection through two major mechanisms: (1) there would be the direct competition between commensal bacteria and pathogens since they require similar ecological niches, including consumption of nutrients, release of bacteriocins or proteinaceous toxins and altering host environmental conditions (such as pH value) [13,14]; (2) the commensal microbiota could also inhibit the growth of the pathogens indirectly by activating the defense responses in the host that can be enhanced by the commensal microbiota through promoting mucosal barrier function, such as eliciting the production of antimicrobial peptides and IgA [15,16,17]. Moreover, commensal microbiota were known to activate host immune responses by maintaining the homeostatic levels of cytokines or inducing the development of certain cell types with protective effects [18]. It has also been reported that commensal microbiota and virulence specific IgG could work together to eliminate the phenotypically avirulent and virulent *Citrobacter rodentium* infection in intestine [19]. Conversely, some diseases would emerge in association with the presence of an abnormal proportion of bacteria. For example, upon disturbance of the commensal microbiota, colonization restriction would be disrupted which may result in the overgrowth of pathogens. It has been reported that Crohn’s disease could be associated with a subnormal proportion of *Bacteroidetes* in the gastrointestinal tract [20,21,22,23], while active celiac disease is caused by excessive *Bacteroidetes* and *Escherichia coli* in the gut [24,25,26] or oral cavity [27]. In contrast, the composition of predominant microbes would fluctuate in response to disease occurrence [28], as well as other environmental perturbations, such as age, geographical locations [29], diet [30], and antibiotic treatment [31]. Therefore, it would be interesting to investigate the dual roles of microbes in the course of diseases.

**Figure 1:**
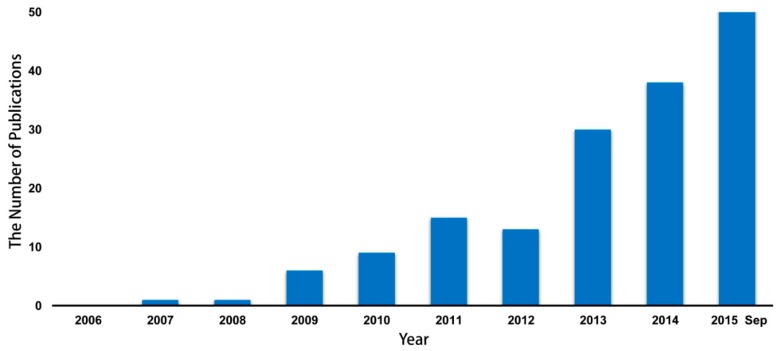
The number of publications reporting the relationship between metagenomics and infectious diseases identified by searching “metagenomics” with “infectious disease”, “contagious disease”, or “transmission disease” as keywords from 2006 to September, 2015.

Recently, the number of studies exploring the correlation between microbiome and the emergence of infectious diseases using metagenomic sequencing has gradually increased around the world (Figure 1) according to the PubMed database. In this review, we provide an overview of the recent discoveries in the connection between the alteration of microbiome in patients and the prevalent infectious diseases, including infections with human immunodeficiency virus/acquired immune deficiency syndrome (HIV/AIDS), tuberculosis, influenza, and hepatitis B virus.

## 2. The Alteration of Microbiome in HIV/AIDS Patients

One of the distinctive features of HIV infection is the depletion of CD4+ T-lymphocytes [32]. Normally, development of AIDS may effectively destruct the immune system of the host. As a result of the loss of CD4+ T cells, HIV/AIDS patients without antiretroviral therapy (ART) are highly susceptible to infections by different microbes in the natural environment [33]. Microbial infection can be regarded as a hallmark of AIDS, which causes significant morbidity and mortality [34,35]. In order to improve the understanding of the disease management, several projects aimed to portray the profile of microbial genetic elements in HIV/AIDS patients and healthy adults have been conducted in different anatomical sites, such as the gastrointestinal (GI) tract, genital tract, and blood stream.

### 2.1. Alteration of the Microbiome in Gastrointestinal Tract

The microbiota in the gastrointestinal tract has been demonstrated to play a major role in the maintenance of health through immune response, nutrition, or metabolism [36]. In HIV/AIDS patients and SIV-infected rhesus monkeys, the microbial translocation from GI tract to systemic circulation is a major reason for the chronic immune activation through intestinal epithelial damage [37,38].

In the rhesus macaque model, although the microbiome in the colon has been reported to be altered between healthy and diseased animals with colitis using 16S rRNA sequencing, it could not be associated with the SIV infection [39]. Actually, several studies showed that bacterial components of the gut microbiome would remain stable in the course of SIV infection in macaques and gorillas [39,40,41,42]. Considering that the 16S rRNA sequencing could not be used to identify viruses, another study aimed to define the enteric virome during SIV infection in non-human primates has been conducted subsequently with metagenomic sequencing. The enteric virome was expanded significantly upon pathogenic SIV infection and 32 novel enteric viruses were identified. As mentioned above, there was no association between enteric bacteria and pathogenic SIV infection, suggesting that the virome was important in the course of the disease [40].

Unlike the situation in SIV-infected monkeys, a previous study indicated that there were high levels of *Enterobacteriales* and *Bacteroidales* in HIV/AIDS patients, which correlated with the depletion of CD4+ T cells and the immune activation [43]. Another recent metagenomic report [44] also showed the enrichment of *Enterobacteriaceae* family and the depletion of *Bacteroidia* class in HIV-infected subjects, which was associated with immune disorder and inflammation symptoms. Meanwhile, Dinh *et al.* found a similar phenomenon in HIV-infected subjects receiving ART [45]. Moreover, Gori *et al.* reported the predominant opportunistic pathogens in fecal flora of HIV-infected subjects. *Candida albicans* and *Pseudomonas aeruginosa* were over-represented in the early stage of the infection. On the other hand, the abundance of protective bacteria, such as *Bifidobacteria* and *Lactobacilli*, were decreased when compared with healthy persons [46]. Later, they provided the prebiotic oligosaccharide mixture in ART-naïve HIV-infected subjects and observed an improved microbial composition. The *Bifidobacteria* was increased while the *Clostridium coccoides/Eubacterium rectale* cluster and *Clostridium lituseburense/Clostridium histolyticum* group levels were decreased. Correspondingly, the CD4+ T cell immune activation was reduced while the innate immune response was improved [47]. In a more recent study, Nowak *et al.* [48] compared the compositions of microbiome among normal controls, HIV-infected patients before and after receiving ART by 16S rRNA sequencing. The diversity of microbiome was significantly lower in HIV-infected patients compared with controls, which persisted after receiving short-term ART. Furthermore, the statistical results clearly showed the reduction of diversity could be correlated with immune dysfunction. A similar phenomenon was also observed by Lozupone *et al.* [49]. However, unlike treatment of naïve patients, the microbiome composition of HIV/AIDS patients on long term ART was more closely resembled to that of normal controls. In conclusion, the HIV infection would change the gut microbiome in patients and the microbiome alteration could not be restored by ART alone.

### 2.2. Alteration of the Microbiome at the Rectal Site

Regarding the rectal mucosal site, a greater change of the microbial composition has been reported. There was significant depletion of *Roseburia*, *Coprococcus*, *Ruminococcus*, *Eubacterium*, *Alistipes*, *Lachnospira*, and enrichment of *Fusobacteria*, *Anaerococcus*, *Peptostreptococcus*, and *Porphyromonas* in HIV-infected persons without antiretroviral therapy compared to normal controls. A similar, but less significant, phenomenon can be observed in the HIV-infected subjects receiving ART [50]. Another recent study was conducted to characterize the microbiome at the terminal ileal and colonic mucosal regions simultaneously [51]. The results confirmed the differentiation between HIV patients and normal people with the rises of *Enterobacteriaceae* and *Prevotella* [52]. The significant increase in *Prevotella* could also be observed in the colon biopsies from the chronic and untreated HIV-infected subjects with the decrease in *Bacteroides* [53]. All these studies suggested that HIV infection would incite a greater representation of pro-inflammatory microbiota in the GI tract than that in healthy subjects, and therefore promote the loss of CD4+ T cells as well as the replication of the virus. Moreover, considering the fact that dysbiosis of the microbiome is associated with increased *Proteobacteria*, Burgener *et al.* hypothesized that HIV-altered mucosal bacteria could directly damage the mucosal barrier associated with microbial translocation [54]. In the future, it would be interesting to investigate the outcomes of the replacement of protective microbial composition with harmful pro-inflammatory microbes.

### 2.3. Alteration of the Microbiome at the Genital Tract

The human vaginal microbiota also plays an important role in the defense against HIV invasion. It has been reported that the sexual transmission rate of HIV from males to females is quite low [55]. Part of the reason is the efficient barrier lining the female genital tract. Specifically, the vaginal microbiota living on the mucus layer help inactivate the virus by secreting H_2_O_2_ or decreasing the pH value of the environment [56]. However, alterations of diet, inflammation, menstrual cycle, and infection with other viruses (HPV (Human papillomavirus) or HSV (Herpes simplex virus) [57,58], *etc.*) will affect the composition and activity of the vaginal microbiota, which may enhance the chance of HIV infection. Among these risk factors, bacterial vaginosis in the genital tract has been considered as a major one and has been investigated worldwide [59]. Bacterial vaginosis is characterized as the reduction of *Lactobacillus* populations and the enrichment of anaerobes, which is also associated with enhanced pro-inflammatory signaling [60]. Schellenberg *et al.* have conducted a study in HIV-exposed seronegative women to find out whether the healthy vaginal microbiota will contribute to HIV resistance [61]. Interestingly, the diagnosis of bacterial vaginosis was similar between HIV-exposed seronegative women and HIV-negative control. Conversely, HIV-infected subjects showed greater possibility to be diagnosed with bacterial vaginosis, indicating the resistant role of the healthy vaginal microbiota. This observation was further confirmed in the study conducted by Hummelen *et al.* clarifying the microbial compositions of women living with HIV using 16S rRNA sequencing [62]. The data were clustered into eight groups according to the similarity of bacterial compositions. In only two of these clusters, *Lactobacillus iners* or *Lactobacillus crispatus*, which were highly correlated with normal microbiota, was predominant. In contrast, in the other four clusters, *Prevotella bivia*, *Lachnospiraceae*, or a mixture of other species, which were highly correlated with bacterial vaginosis, were over-represented. In general, as concluded by Salas *et al.* [63] and Cone *et al.* [64], the vaginal dysbiosis or polymicrobial microbiota would increase the risk of HIV transmission. However, the scenario was quite different in some other studies. Mehta *et al.* have investigated the vaginal bacterial compositions in normal controls and patients with different HIV infection statuses (stable and progressive) with multiple clinical visits [65]. Although six clusters could be classified based on the bacterial compositions, there was no correlation between the clusters and HIV statuses. Another recent study exploring the variations of genital microbiota between different geographic regions [66] found that the general genital microbiota was similar among different groups, although they have confirmed the lower abundance of *Lactobacillus crispatus* in most HIV-infected women. Thus, the inconsistent presentations in different scenarios indicated that there are other underlying mechanisms influencing the outcomes. Besides the comparison of bacterial components between HIV/AIDS patients and normal controls, it has been reported that the virome in the genital tract may be altered by HIV infection: intensely diversified HPV types together with two novel HPV types were identified after HIV infection [67].

### 2.4. Alteration of the Microbiome in the Blood, Semen and Brain

Previously, human plasma in immuno-competent individuals is assumed to be sterile and, therefore, no microbes except viruses should be present. This might not be the case in immuno-compromised hosts, as exemplified by the development of opportunistic infection in HIV-infected patients who are progressing to AIDS [33]. The severe complications of such infection can be preceded by asymptomatic bacteremia or viremia, the characterization of which would be useful for supporting clinical diagnosis and introduction of prophylaxis. In one previous study [68], the microbial elements in the blood of HIV-infected patients without receiving ART compared with normal subjects were reported. As expected, the amount of bacterial DNA was lower in healthy controls, but huge amounts of bacterial DNA could be identified in the patients with *Pseudomonadales* as the predominant component of the microbiome. Furthermore, the bacterial elements similar to the microbes living in the GI tract were found, implying the potential microbial translocation. In this study, the virome between HIV-infected patients and normal controls has also been evaluated. Surprisingly, the over-representation of bacteriophages and endogenous retroviruses were identified in patients. The presence of bacteriophages in HIV/AIDS virome may in other way reflect the presence of bacteria in patients’ plasma while the identification of HERV (Human endogenous retroviruses)-K fragments implied the reactivation of endogenous retroviruses by HIV. In another study [69], Merlini *et al.* evaluated the relationship between the microbial translocation in peripheral blood and the poor response after receiving ART in HIV patients. The 16S rRNA sequencing results indicated the translocating bacterial microbiota was enriched in *Enterobacteriaceae*, without the presence of probiotic *Lactobacillus* spp. both prior and after 12-month ART. This suggested the incompetence of ART in preventing the microbial translocation and purging the circulating microbes. Furthermore, the plasma virome have also been altered by HIV infection in the patients from USA or Uganda [70]. Notably, the HIV, HCV (Hepatitis C virus), HBV, GBV-C (GB virus C), HERV, and increasing proportion of anellovirus have been detected in all HIV-infected subjects. Moreover, the profiles of the microbiome in HIV infected subjects and other controls have also been portrayed in the brain, which is another organ considered to be sterile, previously [71]. Unlike the results from the blood, surprising results showed that microbial composition was predominated by α-*proteobacteria* in all samples regardless of immune status and further analysis revealed the existence of bacteriophage and HHV (human herpes viruses). In addition to blood, HIV could also be transmitted through semen. An interesting study using 16S rRNA sequencing suggested that HIV infection would also change the microbiome in semen. *Mycoplasma* species were found to be dominant after HIV infection, compared to the more abundant *Ureaplasma* in controls, while the semen microbiome diversity and richness would be decreased by HIV infection [72].

### 2.5. Alteration of the Microbiome in Oral Cavity and Airway

In addition, the alteration of the microbiome on the surface of the oral cavity and airway has also been found to be associated with HIV infection. Dang *et al.* have observed a shift of microbial composition in the lingual region, which was related to the viral load in early-stage HIV patients [73]. Another study also revealed the alterations of microbiome in the lung of HIV-infected patients. *Tropheryma whipplei* was predominant but its abundance could be reduced by ART [74]. In contrast, Iwai *et al.* found that in HIV-infected patients the population of *Proteobacteria* was specifically expanded on the airway compared to the oral microbiome [75]. Furthermore, pathogenic organisms have also been proved to be increased on the airway upon HIV infection, such as *Klebsiella pneumoniae* and *Pseudomonas* spp., implying their potential role in recurrent pneumonia. By comparing the lung microbiome from HIV-infected pneumonia patients between US and Uganda, Iwai *et al.* further confirmed that *Pseudomonas aeruginosa* was the most common bacterial pulmonary pathogen in Uganda HIV-infected patients [76]. Moreover, the difference of pneumonia-associated airway microbiome in different geographic regions may indicate the mechanisms accounting for the different clinical outcomes. In another recent report, by comparing the oral and lung microbiome in HIV infected patients and normal controls conducted by Beck *et al.* using 16S rRNA sequencing data [77], they found that the composition of the microbiome were similar and irrelevant with HIV infection or CD4+ T cell count. In contrast, Kistler *et al.* also compared the oral microbiome between HIV-infected and uninfected individuals using saliva and dental plaque samples instead of oral washes by 16S rRNA sequencing [78]. Although it was found that the compositions of microbiome from saliva and dental plaque were quite different, the overall similarity could also be identified in each type of sample regardless of HIV infection. Additionally, the bacterial composition, the fungal compositions from oral wash, induced sputa, and bronchoalveolar lavages were compared between HIV-positive and negative individuals by 18S and internal transcribed spacer sequencing [79]. Most interestingly, it was found that the lung mycobiome were different according to HIV status while *Pneumocystis jirovecii* was over-represented in the lung of HIV-infected patients. Furthermore, it is believed that more results would be achieved through the ongoing Lung HIV Microbiome Project (LHMP) in the future [80].

Although all results mentioned above contributed to better understanding of microbes at different sites in HIV/AIDS disease progression, the comprehensive and conclusive knowledge is still lacking. In order to improve the management of HIV/AIDS progression, new challenges are to understand the underlying reason for the occurrence of microbiome alteration as well as its clear role in regulating the immune response in the host. Moreover, the influences of ART and adjuvant therapy by restoring commensal microbiota in HIV/AIDS treatment need further investigation.

## 3. The Alteration of Microbiome in Patients with Tuberculosis

Similar to gastrointestinal tract, the human respiratory tract is another region which is heavily exposed to microorganisms, including influenza virus, *Mycobacterium tuberculosis*, and respiratory syncytial virus. All exogenous microbial invasions will cause major morbidity and mortality. With the advancement of the sequencing technique, it is now possible to capture the alteration of the microbiota profile in the host in response to the infection of these dangerous pathogens.

Tuberculosis is indeed another worldwide health concern. It is estimated that one-third of the world’s population is infected with the *Mycobacterium tuberculosis* in the latent form. After several studies reporting the possible relationship between the presence of *Helicobacter pylori* in the human body and increased resistance to tuberculosis [81], the aim of many studies has diverted from *Mycobacterium tuberculosis*, itself, to the altered microbial compositions within tuberculosis patients.

Competing with traditional culturing methods, the next-generation sequencing technology has been used in the analysis of the microbiome from the sputum samples so as to provide a more comprehensive view in reflecting the microbiota from the lower respiratory tract. Cui *et al.* have observed more diverse compositions of the microbiome in tuberculosis patients which formed a clear clustering pattern when compared to the healthy controls [82]. The dominant phyla of *Firmicutes, Bacteroidetes*, and *Proteobacteria*, which accounts for 70% of the microbiome, could be identified in both patients and healthy controls and these results were also observed in another study conducted by Cheung *et al.* [83]. Cui *et al.* have also found several foreign bacteria unique to the tuberculosis patients at the genera level, such as *Stenotrophomonas, Cupriavidus*, and *Pseudomonas*. On the other hand, Cheung *et al.* proposed a different list of core genera in tuberculosis sputum samples, including *Actinomyces*, *Fusobacterium*, and *Leptotrichia*, while *Streptococcus*, *Neisseria*, and *Prevotella* were the most predominant genera in both patients and controls. Furthermore, Wu *et al.* have correlated the compositions of the microbiome with the different statuses of tuberculosis, as well as the prognosis [84]. They found that the ratios of *Pseudomonas* to *Mycobacterium* from recurrent tuberculosis patients or treatment failure patients were higher than that in newly identified tuberculosis patients. Moreover, in a recent study, Botero *et al.* sequenced the microbiome from sputum, oropharynx, and nasal respiratory tract samples. The different bacterial and fungal compositions between patients with pulmonary tuberculosis and healthy controls could only be identified in oropharynx samples [85]. Among all these studies, the compositions of the microbiome were not consistent with each other to some extent. It might be caused by the different criteria and standards in selecting and analyzing samples. In addition, all studies associated with tuberculosis were conducted by 16S rRNA sequencing, which means that the viral and other eukaryotic components could not be revealed. In future investigations, more comprehensive profiles should be elucidated by the shotgun approach using deep sequencing.

Furthermore, although *Mycobacterium tuberculosis* is an important human pathogen, the diagnosis remains challenging. In addition to clarifying the microbiome in the lower respiratory tract, Winglee *et al.* investigated the relationship between tuberculosis and the gut microbiota in mice. Fluctuation of the microbiome could be observed after the infection of *Mycobacterium tuberculosis* [86]. It is worthwhile to investigate whether similar results could be obtained in humans, considering the important role of gut microbiota in relation with immune response.

## 4. The Alteration of Microbiome in Patients Infected with Influenza

The past fourth influenza pandemic in 2009 has alerted people that the influenza virus is still a major threat to human beings. One of the major risk factors responsible for the high mortality in the previous pandemics is the secondary bacterial pneumonia elicited by *Streptococcus* and *Pneumococci* [87]. However, unlike tuberculosis infection, the influenza infection is an acute infection. Therefore, in earlier studies the major application of the new sequencing techniques is to identify viral species in influenza patients. With the growing understanding of the important roles of microbiota in the upper respiratory tract, several groups have conducted studies aiming to investigate the association between microbial composition and the secondary infection using the next-generation sequencing technology.

One year after the 2009 pandemic caused by H1N1 influenza, Greninger *et al.* have characterized the viral metagenomes from the samples of nasopharyngeal swabs in patients [88]. They have also analyzed the results from the upper respiratory microbiota. It is a little surprising that the presence of co-pathogens was very low in all samples considering that the *Streptococcus pneumoniae* has been reported to play an important role in this pandemic [89]. In another study, it appeared that there was no significant difference of the *Streptococcus* species in the H1N1-infected and uninfected pneumonia patients, as well as other common causative agents related to bacterial pneumonia [90]. Their microbial profiling results between H1N1-infected and uninfected groups have been shown at phylum, family, and genus levels while the results at phylum level and family level were somehow consistent with that from Chaban *et al.* [91]. In particular, *Firmicutes* and *Proteobacteria* phylum, as well as the *Moraxellaceae* family could be identified as the predominated bacteria in the patients with H1N1 influenza in both reports. It suggested the potential correlation between these bacteria and influenza infection. Lately, Yi *et al.* conducted another studies to investigate the alteration of microbiome in upper respiratory tracts after the viral respiratory infections including the influenza using 16S rRNA sequencing. It showed that *Streptococcus* species were dominated in healthy subjects while *Haemophilus* or *Moraxella* were enriched in the patients [92]. However, the significant association between respiratory viruses and microbiome in upper respiratory tracts could not be identified. Based on the results of gene clustering and ontology, this study proposed that the species with increased abundance in H1N1-infected patients were enriched with chemotaxis and flagellar assembly genes [90] while these genes were absent in species with decreased abundance. In other words, the functions of unique genes in microbes predominant in influenza patients may play a role in helping the microorganisms to gain access to the lower respiratory tract. Nevertheless, the exact roles of these microorganisms in the pathogenesis of influenza deserve future investigation.

## 5. The Alteration of Microbiome in Patients Infected HBV

Hepatitis B is a severe liver infection caused by the infection of hepatitis B virus (HBV). There is a high prevalence of HBV infection in sub-Saharan Africa and East Asia, which will cause chronic liver disease leading to high risk of death from liver cirrhosis and cancer. Recently, the next generation sequencing techniques has also been applied to study the correlation between HBV infection and microbe population in the host. Interestingly, it was commonly shown that intestinal microbiota would be altered upon liver diseases [93]. Moreover, the importance of microbiota from GI tract has been well illustrated in the development of liver cirrhosis driven by bacterial translocation and the subsequent pro-inflammatory effects [94,95].

In a recent study, Law *et al.* have conducted the metagenomic sequencing in the plasma from patients with different liver diseases, including patients with chronic hepatitis B and C infection, autoimmune hepatitis, and non-alcoholic steatohepatitis. As expected, the results showed that the hepatitis virus has taken the predominant position upon HBV or HCV infection while the alterations of other viruses was quite limited, indicating that the structure of plasma virome, other than HBV or HCV, may not be changed [96]. Another study revealed that the diversity of fungal microbiota, also known as mycobiome, was related with disease progression of patients infected with HBV [97]. The general trends showed that the fungal diversity would be enhanced in more severe level of HBV infection. Similarly, based on the results from 16S rRNA sequencing and shotgun metagenomic sequencing, respectively, Wei *et al.* and Chen *et al.* reported that significant changes in bacterial compositions of the GI tract could be associated with HBV-induced cirrhosis [98,99]. The results of these two independent studies were quite consistent at the family level: the enrichment of *Enterobacteriaceae*, *Veillonellaceae* and *Streptococcaceae*, which were considered as pathogenic, combined with the reduction of *Bacteroidaceae* and *Lachnospiraceae*, which were beneficial for the host. The prevalence of *Enterobacteriaceae* and *Streptococcaceae* can also be confirmed by other clinical studies, which indicated that *Escherichia coli* and *Streptococcus* were the main causes of bacterial infection with cirrhosis [100]. These results implied potentially important functions of these bacteria associated with HBV-induced cirrhosis. However, it should be noted that Chen *et al.* have also compared the difference of the microbiome in HBV-related and alcohol-related patients with liver cirrhosis [99]. The similar structure of microbial communities in these two groups of patients suggested that HBV, itself, may not be involved in the alteration of the host microbiome during liver cirrhosis.

## 6. Conclusion

**Figure 2:**
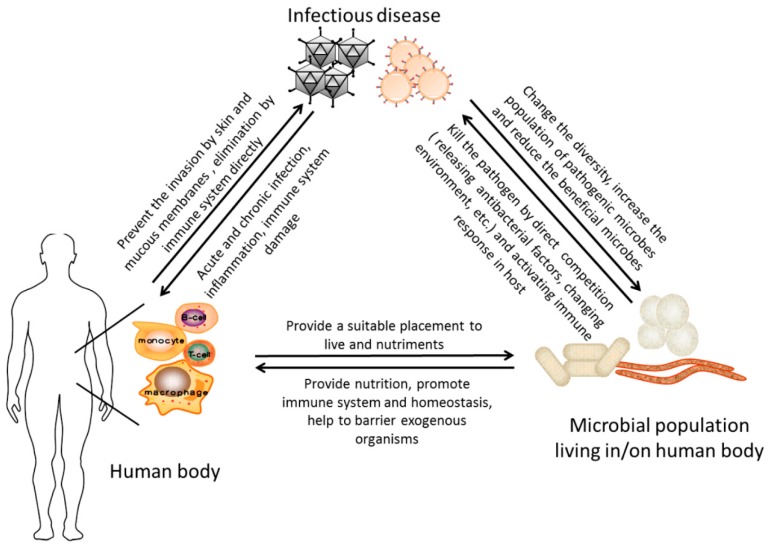
The schematic diagram of the relationship among the human body, the host microbiome, and infectious disease.

It has been noticed that HIV infection is the major focus of the host–microbiota interaction studies partly because rapid change of the immune system in the host will bring remarkable alteration of the microbiome. Additionally, of all the worldwide concerned infectious diseases mentioned above, investigations in the alteration of microbiome have also been conducted upon the infection with HPV, rotavirus, *Salmonella*, *Clostridium difficile*, and enteropathogenic *Citrobacter rodentium* [101,102,103,104,105,106,107,108], or to clarify its relationships with immune responses of oral vaccine in infants [109]. It is no doubt that the new sequencing techniques have been widely used to provide a more comprehensive understanding of the relationship among infectious diseases, the host microbiome and the human body. A brief summary of the relationship were described in Figure 2. Based on the current understanding, some pre-clinical applications modifying the microbiota in the host have been launched, consequently. The basic idea is to recover the mutually beneficial species in the host and curb the secondary injury. The probiotics (live microorganisms which provide health benefits) and prebiotics (non-digestible chemicals selectively induce growth of beneficial bacterial species) have been applied in different infectious disease therapies with inspiring results [47,110,111]. The probiotics may express antimicrobial compounds or changing the gut environments to provide protection while prebiotics can also exert the effect on modulation of the gut homeostasis to eliminate or reduce the growth of bacterial pathogens. As mentioned above, because HIV/AIDS patients were known to have a compromised gut barrier function, as well as the depletion of *Bifidobacterium* and *Lactobacillus* species, Gori has conducted a clinical trial by supplementing three oligosaccharides to reshape the gut microbiota. The results exhibited an improved immune function in HIV patients without receiving ART [47]. A similar study showed the complete inhibition of the pathogenic bacterial growth through a probiotic yogurt [110]. It was suggested that the *Lactobacillus rhamnosus* GR-1 used in this study adhered to the mucosal site to inhibit the microbial translocation and reconstruct the mucosal barriers. This strategy has also been applied in SIV-infected pigtail macaques receiving ART [112] and HIV-infected infants [113], and both studies showed encouraging results with reconstitution of CD4+ T cells. However, some unsuccessful experiments indicated that not all recipes of pro- and prebiotics would exert the effect on alleviation of the complications associated with HIV infection [114]. In addition to pre/probiotics, there were some other strategies tested to modulate gut homeostasis, such as blocking the bacterial pathogenic production of chemicals [115,116,117] or bioactive substances [118,119,120].

In retrospect of the application of high-throughput sequencing methods to clarify the mutual interaction between infectious pathogens and the human microbiome, we noticed that there were an increasing number of relevant articles using the metagenomic sequencing method. We believed that the metagenomic sequencing method exhibited several advantages compared with 16S rRNA sequencing, which would be a more powerful technique for future studies in this area. As mentioned in the introduction, the metagenomic sequencing method could provide more useful information about the eukaryotic organisms and viruses other than bacteria. In addition, the previous concern about shotgun metagenomic sequencing that the sequencing depth could not compare with 16S rRNA sequencing would be alleviated with the reduced cost of next generation sequencing and the computational expense [121]. Moreover, the metagenomic sequencing method would not only characterize the taxonomical diversity of the microbiome but also predict the functional diversity of a community quantified by annotating metagenomic sequences with functions [122]. Furthermore, a recent report indicated that the growth dynamics of gut microbiota could be profiled from a single metagenomics sample by determining the ratio of reads near the replication origin and that near the terminus, reflecting whether the bacteria was in an “active” phase, as well as its correlation with various diseases [123]. Therefore, we believe that the metagenomic sequencing method would be more widely applied in detecting the interaction between infectious diseases and the host microbiome in future.

In addition, some shortcomings still exist in the development of exploring the interactions between infectious disease and microbiome using next generation sequencing techniques. One of the problems are the inconsistent results from different studies concerning the same situation. Since the alterations of the microbiome are calculated from statistical results in a group of subjects with similar conditions, it requires stricter criteria for categorizing subjects with more detailed clinical information. Meanwhile, it has been reported that traditional culturing techniques are still more superior to the high-throughput sequencing method in some cases [124], suggesting that there is still much room for further improvement of sequencing methods and data analysis strategies.

Nevertheless, the metagenomic analysis is poised to be critical to characterize microbial compositions in humans, as well as the potential relationship between microbiome and pathogenesis of hazardous infectious diseases. The aim of future studies is not only portraying the microbiota pattern, but also clarifying the changes in immune response and metabolic functions brought by the alteration of disease-associated microbes. These studies will certainly require the seamless integration of knowledge from microbiology, bioinformatics, immunology, cell biology, and ecology.
